# A comparative study of anxiety symptoms in Chinese and Rwandan adolescents: a cross-cultural measurement invariance study of the GAD-7 scale

**DOI:** 10.3389/fpsyt.2025.1571753

**Published:** 2025-05-09

**Authors:** Lisa Cynthia Niwenahisemo, Qi Zhang, Wo Wang, Dan-dan Geng, He-yan Xu, Jin-hui Hu, Ling-li Ma, Jian-yu Tan, Yi-ting Kong, Su Hong, Li Kuang

**Affiliations:** ^1^ Department of Psychiatry, the First Affiliated Hospital of Chongqing Medical University, Chongqing, China; ^2^ Psychiatric Center, the First Affiliated Hospital of Chongqing Medical University, Chongqing, China; ^3^ Department of Psychiatry, the University-town Hospital of Chongqing Medical University, Chongqing, China; ^4^ Department of Nursing, the First Affiliated Hospital of Chongqing Medical University, Chongqing, China

**Keywords:** generalized anxiety disorder-7 (GAD), GAD-7 scale, cultural differences, adolescent mental health, measurement invariance, cross-cultural comparison, psychometric validation [LN1]

## Abstract

**Introduction:**

Cultural factors and assessment methods significantly influence how anxiety symptoms are expressed and reported. However, few cross-cultural studies have employed culturally appropriate and validated tools, and even fewer have provided substantial comparisons across different groups with diverse cultural backgrounds. This study aimed to assess the measurement invariance of the GAD-7 scale across Chinese and Rwandese adolescents, enabling reliable cross-cultural comparisons.

**Methods:**

This study included 2017 Chinese adolescents and 1813 Rwandan adolescents. Cronbach’s alpha, exploratory factor analysis (EFA), and multiple group confirmatory factor analysis (MGCFA) were used to assess the validity of the GAD-7 scale across the two groups. Measurement invariance testing was employed to investigate cross-cultural equivalence.

**Results:**

The GAD-7 demonstrated good psychometric properties. CFA supported a one-factor model for the GAD-7 in both samples, though model fit indices varied. Measurement invariance testing confirmed configural and metric invariance but found partial scalar invariance. A latent mean comparison indicated a trend toward higher anxiety levels in Rwandan adolescents compared to Chinese adolescents, though the difference was not statistically significant (z = 0.02, d = 0.033, p = 0.98).

**Discussion:**

The GAD-7 showed reliability in measuring generalized anxiety in both Chinese and Rwandese adolescents, confirming its cross-cultural construct validity. However, partial scalar invariance suggests that while the GAD-7 effectively detects anxiety symptoms, the severity of reported symptoms may not be directly comparable across cultures due to response patterns and possible linguistic factors. These findings highlight the importance of culturally sensitive instruments for accurate anxiety assessment and expand evidence on reliable symptom screening and treatment monitoring across diverse populations.

## Introduction

Adolescence is a critical developmental period characterized by significant biological, psychological, and social transformations (1). This stage is particularly vulnerable to the emergence of mental health challenges, with anxiety disorders being among the most prevalent and debilitating conditions affecting adolescent well-being worldwide (2). Epidemiological studies estimate that 15% to 30% of adolescents globally experience clinically significant anxiety symptoms, with notable variations across regions and populations (3, 4). For instance, while anxiety disorders affect 5% to 10% of adolescents in sub-Saharan Africa, prevalence rates rise to 18.6% among teenage girls in Tanzania and 20.4% in Rwanda. In contrast, Chinese adolescents exhibit even higher rates, with approximately 30% reporting anxiety symptoms (5–8). These disparities may reflect true differences in prevalence or methodological challenges in cross-cultural mental health research, including variations in diagnostic sensitivity, cultural expression of symptoms, and measurement validity (9–11). A critical methodological consideration in cross-cultural comparisons is the establishment of measurement invariance, which ensures that observed differences in mental health outcomes reflect true variations in the underlying construct rather than artifacts of measurement bias (12).

Measurement invariance is typically assessed through confirmatory factor analysis (CFA), which evaluates three hierarchical levels: configural invariance (equivalent factor structure), metric invariance (equivalent factor loadings), and scalar invariance (equivalent item intercepts) (12). Without establishing measurement invariance, cross-cultural comparisons of mental health constructs risk being confounded by cultural differences in symptom interpretation and reporting.

The Generalized Anxiety Disorder-7 (GAD-7) scale, a widely used instrument based on DSM-IV diagnostic criteria, has gained recognition for its brevity, reliability, and validity in assessing anxiety symptoms across diverse populations (13–15). However, despite its widespread adoption, questions remain regarding its cross-cultural applicability (16, 17). While the original validation study by Spitzer et al. (14) proposed a unidimensional factor structure, subsequent research has identified variations in factor structures across cultures, including evidence for a two-factor model distinguishing somatic and cognitive symptoms (18). Furthermore, studies have reported partial measurement invariance and differential item functioning (DIF) for specific GAD-7 items, particularly those related to nervousness and excessive worrying, suggesting cultural influences on symptom interpretation (19, 20). These findings underscore the need for rigorous evaluation of the GAD-7’s cross-cultural validity, particularly in understudied populations.

Despite growing interest in cross-cultural mental health research, most studies have focused on Western populations, leaving significant gaps in our understanding of the GAD-7’s applicability in non-Western contexts, particularly in comparisons between Asian and African populations. To bridge this gap, the present study examines the cross-cultural measurement invariance of the GAD-7 in adolescent samples from Rwanda and China, two culturally distinct yet understudied populations. Data were collected from Shapingba District in Chongqing, China, and Gasabo and Kicukiro Districts in Kigali, Rwanda. These regions were selected for their socioeconomic and cultural diversity, as well as their representation of urban, suburban, and rural populations. Chongqing, a megacity with over 31 million residents, encompasses a wide range of socioeconomic backgrounds, while Kigali, Rwanda’s capital, serves as a cultural and educational hub, drawing students from across the country.

Building on prior studies (21, 22), we hypothesized that the GAD-7 would demonstrate a unidimensional structure in both populations and exhibit measurement invariance across cultural groups. Additionally, we aimed to compare latent mean levels of anxiety symptoms between Rwandan and Chinese adolescents, contingent on the establishment of scalar or partial scalar invariance. This study contributes to the growing body of literature on cross-cultural mental health assessment by providing empirical evidence on the GAD-7’s validity in understudied populations, thereby enhancing its utility for global mental health research and practice.

## Methodology

### Participants

The study included 3,830 adolescents aged 12 to 18, recruited from middle and high schools in Shapingba District, Chongqing Municipality, China (n=2,017), and Gasabo and Kicukiro Districts in Kigali, Rwanda (n=1,813). In Shapingba District, which comprises 17 junior high schools and 11 senior high schools, a stratified random sampling approach was employed based on urban and suburban areas to ensure sample diversity and representativeness. Schools were stratified by geographical location (urban or suburban), and within each stratum, schools were randomly selected. Specifically, 3 urban schools and 2 suburban schools were chosen to reflect the socioeconomic and cultural diversity of the district. From these schools, at least two classes per grade level were randomly selected, and students within each class were further chosen using a random number table or generator to ensure equal representation.

In Rwanda, a cluster sampling method was utilized due to the geographical distribution of schools to select schools that represented a diverse range of students from rural, urban, and suburban areas across all provinces of the country. Five secondary boarding schools (2 public and 3 private) were randomly selected from a total of 27 schools in Gasabo and Kicukiro Districts. Within each school, classes from both ordinary (grades 7-9) and advanced (grades 10-12) levels were randomly selected using a random number generator. Approximately 2000 students were targeted from each country, with strict adherence to randomization procedures to minimize bias. All participating researchers received comprehensive training to ensure consistency and accuracy. Data collection spanned from 2021 to 2023, and the sample size was calculated using Franklin K.’s formula to ensure adequate statistical power and generalizability.

### Data collection

Data collection methods were tailored to the local context in each country. In China, surveys were administered via a secure online platform, allowing for anonymous submissions. This approach was chosen to align with the high prevalence of internet access and technological literacy in metropolitan Chongqing. In Rwanda, where boarding school students often have restricted access to personal devices, in-person data collection was conducted in classroom settings under the supervision of trained researchers. Surveys were administered in the participants’ native languages (Mandarin for Chinese participants and Kinyarwanda for Rwandan participants) to minimize language-related bias and enhance comprehension.

All participants and their parents or guardians provided written informed consent after being fully informed of the study’s objectives, voluntary nature, and their right to withdraw at any time without consequences. To ensure anonymity, student ID numbers were used as unique identifiers, with no additional personal information collected. Ethical approval was obtained from the College of Medicine and Health Sciences Institutional Review Board of the University of Rwanda (CMHS IRB; No. 465/CMHS IRB/2022) and the Ethics Committee of the First Affiliated Hospital of Chongqing Medical University with research batch number (No. 2020-879).

### Assessment instrument

The Generalized Anxiety Disorder Symptom Severity (GAD-7) Scale, developed by Spitzer (14), was used to assess anxiety symptom severity. The GAD-7 is a well-validated, self-report instrument consisting of seven items that measure symptoms such as nervousness, excessive worrying, difficulty relaxing, restlessness, impatience, and persistent fearfulness (Löwe et al., 2008b; Ruiz et al., 2011). It employs a 4-point Likert scale (0 = not at all to 3 = nearly every day), with total scores ranging from 0 to 21. Cut-off scores of ≥5, ≥10, and ≥15 to indicate mild, moderate, and severe anxiety symptoms respectively (14). For adolescent populations, a cut-off score of ≥7 is recommended based on community-based studies (23). The GAD-7 has demonstrated strong psychometric properties, including high reliability and validity, in both clinical and community settings (18, 24).

### Statistical analyses

Data was analyzed using SPSS (Statistical Package for the Social Sciences) version 26 for descriptive statistics, and IBM AMOS (Analysis of Moment Structures) version 26 for structural equation modeling. Preliminary analyses included checks for missing values, outliers, and normality assumptions. Descriptive statistics were computed to summarize demographic characteristics and GAD-7 within each group. Independent t-tests were used to compare mean GAD-7 scores between Chinese and Rwandan adolescents, while chi-square tests examined gender distribution differences.

To evaluate the psychometric properties of the GAD-7, Pearson’s correlation analysis was conducted to assess item-total correlations, followed by Cronbach’s alpha to determine internal consistency (18). Exploratory factor analysis (EFA) was performed using principal axis factoring with oblique rotation (Promax) to explore the underlying factor structure within each cultural group. The number of factors retained was determined based on scree plot and eigenvalues greater than one.

Confirmatory factor analysis (CFA) was then conducted to test the fit of the GAD-7’s unidimensional model in each cultural group. Model fit was assessed using multiple indices, including the chi-square goodness-of-fit test, Comparative Fit Index (CFI), Tucker-Lewis Index (TLI), and Root Mean Square Error of Approximation (RMSEA). Standard thresholds were applied to determine acceptable model fit (CFI > 0.95, TLI > 0.95, RMSEA < 0.08). Measurement invariance across the Chinese and Rwandan samples was tested using multi-group CFA, examining three hierarchical levels: configural invariance (equivalent factor structure), metric invariance (equivalent factor loadings), and scalar invariance (equivalent item intercepts). Changes in fit indices (e.g., ΔCFI, ΔRMSEA) were used to evaluate invariance at each level as a criterial for invariance test based on Cheung and Rensvold (2000)’s standards. It was proposed that according to their guidelines, a ΔCFI ≤ 0.01 and ΔRMSEA ≤ 0.015 thresholds (25). If scalar or partial scalar invariance was established, latent mean comparisons of anxiety symptoms between the two groups were conducted.

## Results

### Descriptive statistics

This study compared anxiety symptoms among adolescents in China and Rwanda. The sample included 2017 Chinese adolescents (56.7% male, 43.3% female; mean age = 15.35 ± 1.56 years) recruited from middle and high schools in Chongqing, and 1813 Rwandan adolescents (51.2% male, 48.8% female; mean age = 15.80 ± 1.90) from Kigali.

**Figure 1:**
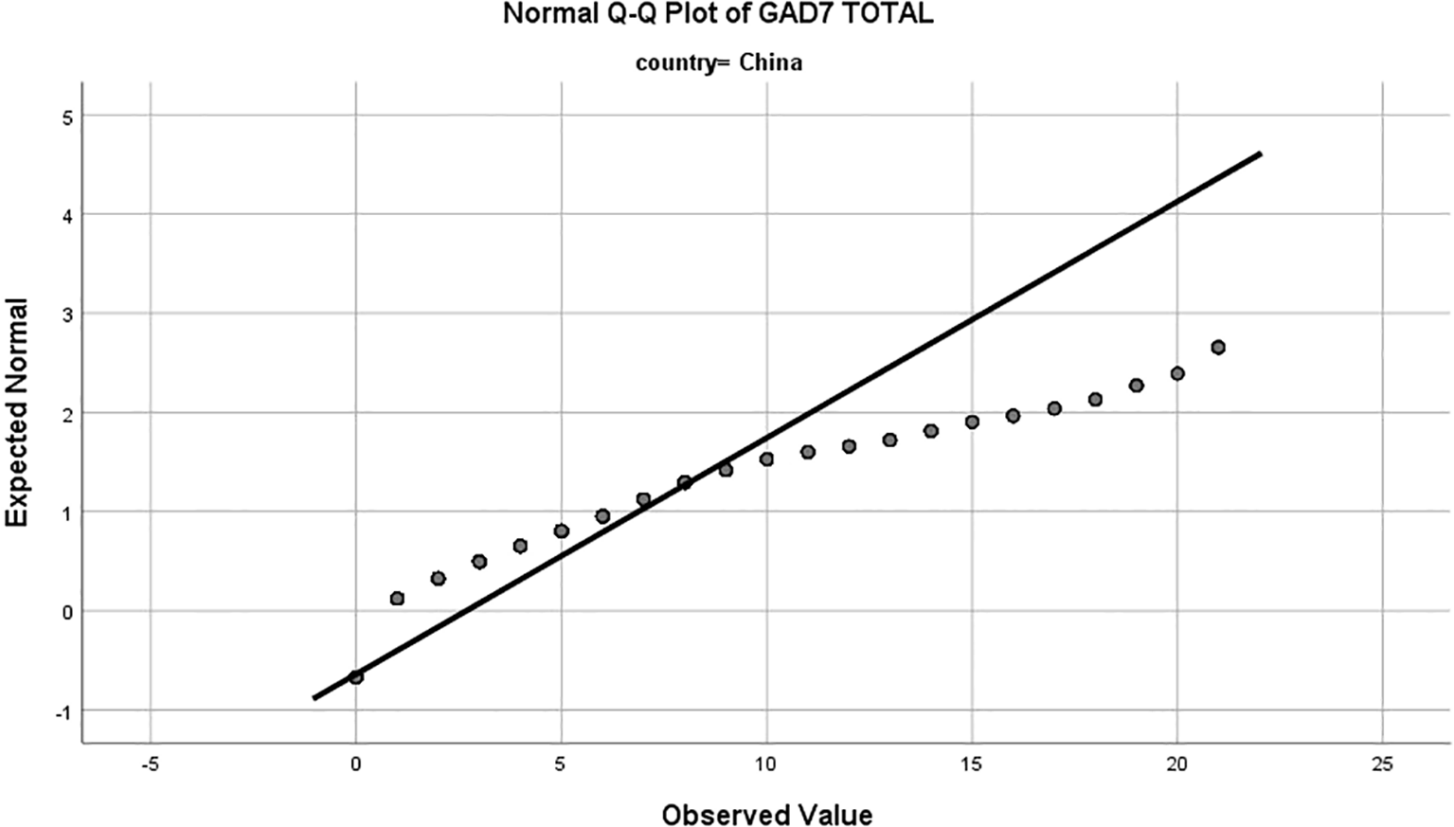
Normal Q-Q plot of Generalized Anxiety Disorder-7 (GAD-7) total scores for Chinese adolescents. The x-axis represents the expected normal distribution of scores under the assumption of normality, while the y-axis represents the observed values of GAD-7 scores. Deviations from the diagonal line indicate departures from normality, with the Chinese sample showing a positive skew, suggesting a higher frequency of elevated anxiety scores.

**Figure 2:**
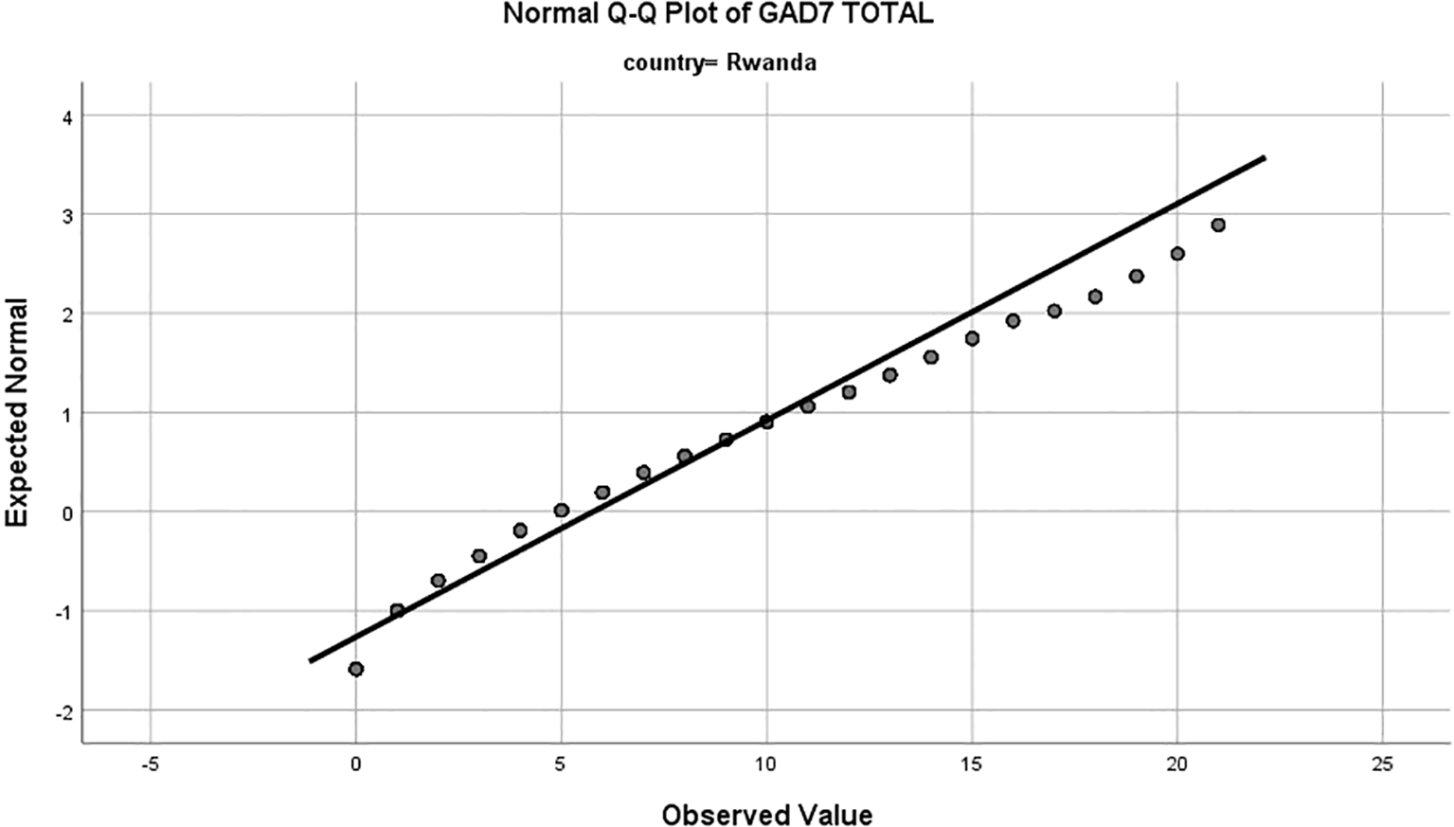
Normal Q-Q plot of Generalized Anxiety Disorder-7 (GAD-7) total scores for Rwandan adolescents. The x axis represents the expected normal distribution of scores under the assumption of normality, while the y-axis represents the observed values of GAD-7 scores. The Rwandan sample displays a distribution closer to normality, albeit with a slight positive skew at higher scores, indicating a tendency toward higher anxiety levels.

Rwandan adolescents reported significantly higher average GAD-7 score (M = 5.7, SD = 4.5) compared to their Chinese counterparts (M =2.6, SD = 4.1), indicating a higher prevalence of generalized anxiety disorder symptoms in the Rwandan sample (52.5% vs. 23.2%, P<0.001; **Table 1**). Examination of the Normal Q-Q plots revealed deviations from a perfect normal distribution for both samples. The Chinese sample exhibited a positive skew, suggesting a higher frequency of elevated anxiety scores, while the Rwandan sample displayed a distribution closer to normality, albeit with a slight positive skew at higher scores. (**Figures 1**, **2**).

**Table 1:** Sample demographic features descriptive statistics.

Variables	Rwandese adolescents		Chinese adolescents		Sample comparison tests		
	N (%)	M (SD)	N (%)	M (SD)	T-test (DF)	χ2 (DF)	Mean difference (95% CI)
Male	928 (51.2)	15.9 (1.9)	1143 (56.7)	15.4 (1.5)		11.56* (1)	
Female	885 (48.8)	15.6 (1.8)	874 (43.3)	15.2 (1.5)			
Age (12-15)	754 (41.6)	13.93 (1.13)	1075 (53.3)	14.06 (0.76)			
Age (16-19)	1059 (58.4)	17.13 (1.01)	942 (46.7)	16.82 (0.73)			
Total number	1813	5.7 (4.5)	2017	2.6 (4.1)			
GAD-7 TOTAL SCORES					21.86* (3828)		3.10 (2.82, 3.37)

N, number of participants, M, sample mean, SD, standard deviation, CI, Confidence interval range, DF, Degree of freedom, χ^2^, Chi-square t*= T-test value (P < 0.05).

### Scale features and baseline model establishment

**Figure 3:**
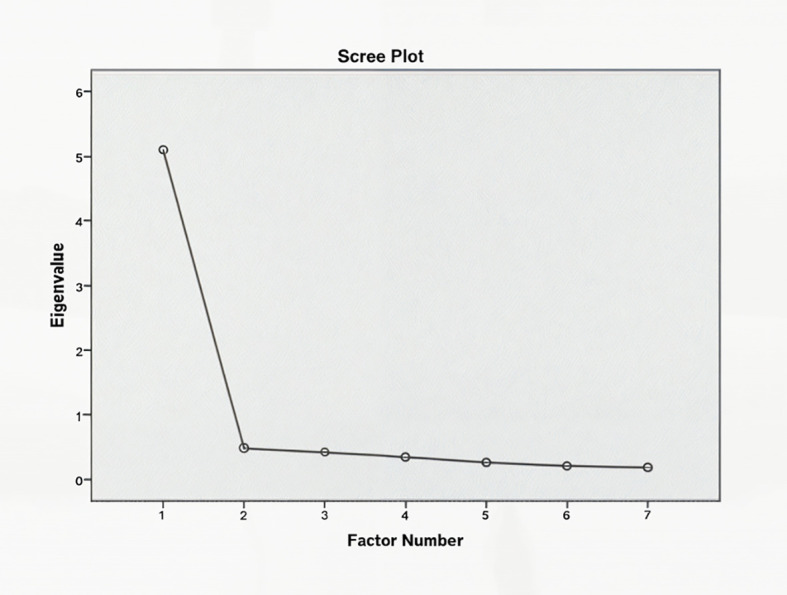
Scree plot for the Generalized Anxiety Disorder-7 (GAD-7) scale in Chinese adolescents. The x-axis represents the factor number, and the y-axis represents the eigenvalues, which indicate the amount of variance explained by each factor. The plot shows a clear elbow at the second factor, supporting a one-factor solution for the GAD-7 in the Chinese sample, consistent with the unidimensional structure of the scale.

**Figure 4:**
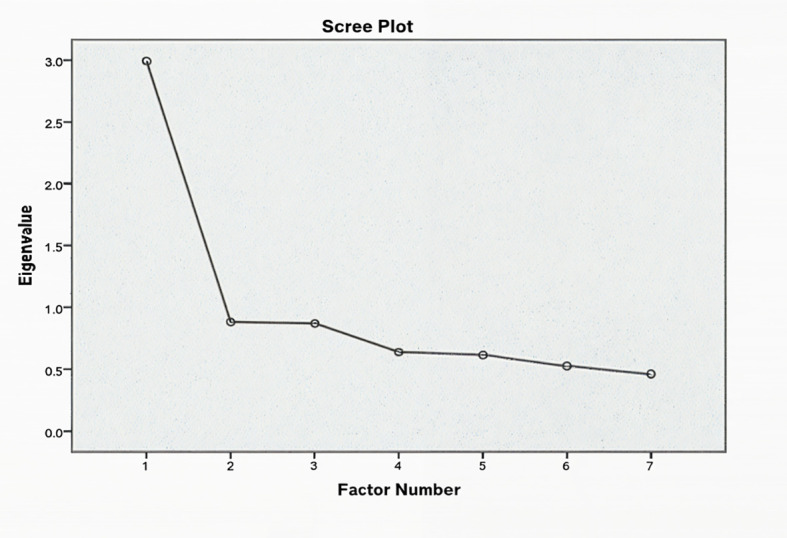
Scree plot for the Generalized Anxiety Disorder-7 (GAD-7) scale in Rwandan adolescents. The x-axis represents the factor number, and the y-axis represents the eigenvalues, which indicate the amount of variance explained by each factor. The plot shows a clear elbow at the second factor, supporting a one-factor solution for the GAD-7 in the Rwandan sample, consistent with the unidimensional structure of the scale.

Scree plots were used to determine the optimal factors structure for the GAD-7. For both samples, a one-factor solution was supported, as evidenced by eigenvalue and scree plots complimenting (**Figures 3**, **4**). The GAD-7 demonstrated strong reliability in both groups (Cronbach’s **α** ≥ 0.70), and all items were retained, as their removal would reduce the scale’s reliability. Item-total correlations fell within the acceptable range (0.30 to 0.70) (26) (**Table 2**).

**Table 2:** GAD-7 reliability and item correlation coefficients.

Items	Rwandese students						Chinese students					
	M	SD	I	C	F	α	M	SD	I	C	F	α
ITEM 1	0.89	1.008	0.680	0.531	0.694	0.736	0.46	0.728	0.859	0.801	0.892	0.927
ITEM 2	0.74	1.018	0.729	0.595	0.752	0.723	0.36	0.705	0.899	0.805	0.866	0.921
ITEM 3	1.10	1.090	0.728	0.582	0.735	0.725	0.46	0.746	0.882	0.831	0.864	0.924
ITEM 4	0.58	0.893	0.609	0.463	0.626	0.750	0.38	0.708	0.881	0.833	0.831	0.924
ITEM 5	0.49	0.863	0.581	0.435	0.587	0.755	0.29	0.619	0.847	0.796	0.825	0.928
ITEM 6	.90	1.076	0.571	0.379	0.519	0.769	0.40	0.722	0.827	0.759	0.787	0.931
ITEM 7	1.08	1.072	0.653	0.484	0.633	0.746	0.34	0.690	0.774	0.694	0.717	0.936
GAD-7	5.79	4.579				0.772	2.68	4.197				0.937

GAD-7, Anxiety symptoms assessment scale; α, Cronbach’s alpha; M, mean; SD, standard deviation; I, Item total correlation; C, Corrected Item total-correlation; F, factor loadings.

### Measurement invariance of the GAD-7

#### Single-group Confirmatory Factor Analysis

Single-group CFA was conducted to assess the fit of the original one-factor model. The Rwandese group showed acceptable model fit indices (CFI = 0.940, RMSEA = 0.08, SRMR = 0.041), while the Chinese group exhibited excellent CFI and SRMR values but a poor RMSEA (0.102). Modification indices suggested correlations between the intercepts of items 2 (“not being able to stop or control worrying”) and 3 (“worrying too much about different things”), items 3 and 4 (“trouble relaxing”), and items 4 and 5 (“being so restress that it is hard to sit still”) in both samples. Accounting for these correlations significantly improved model fit (Δχ^2^ (3) 195.853, P<0.001; **Table 3**).

**Table 3:** Multiple group measurement invariance of GAD-7.

GAD-7	χ2(df)	CFI	RMSEA (90% CI)	SRMR	GFI	TLI	NFI	ΔCFI	ΔRMSEA	Δχ2*(df)
Single group CFA-original factor										
Rwandese students	174.42 (14)	0.940	0.08(0.069, 0.090)	0.041	0.973	0.911	0.936	–	–	–
Chinese students	307.29 (14)	0.974	0.102(0.092, 0.112)	0.025	0.957	0.961	0.973	–	–	–
Single group CFA (θ3, 4 free; θ2, 3 free; (θ4, 5free)										
Rwandese students	63.946 (11)	0.980	0.052 (0.040, 0.064)	0.026	0.991	0.962	0.976	0.040	0.028	110.48
Chinese students	111.44 (11)	0.991	0.067(0.056, 0.079)	0.017	0.984	0.983	0.990	0.017	0.035	195.85*
MGCFA (θ3, 4 free; θ2, 3 free; (θ4, 5free)										
**Configural invariance**	175.42 (22)	0.989	0.043 (0.037,0.049)	.026	0.987	0.979	0.988	–	–	
**Metric invariance**	289.66 (28)	0.981	0.049 (0.044, 0.055)	0.051	0.979	0.972	0.979	0.008	0.006	114.24* (6)
**Scalar invariance**	744.99 (34)	0.949	0.074(0.069, 0.079)	0.047	–	0.937	0.947	0.032	0.025	455.32* (6),
τ7 free	517.93 (33)	0.966	0.062(0.057, 0.067)	0.049	–	0.956	0.963	0.015	0.013	228.26* (5)
τ7, τ3 free	412.46 (32)	0.973	0.056(0.051, 0.061)	0.050	–	0.964	0.971	0.008	0.007	122.79* (4),

GAD-7, generalized anxiety disorder scale-7; SRMR, Standardized root mean square; RMSEA, root mean square error of approximation; NFI, The Normed Fit Index; CFI, Comparative Fit Index; TLI,Tucker-Lewis Index; GFI, Goodness of Fit Index. Δχ2*=chi-square difference is significant at P<0.001; CFA-original factor = confirmatory factor analysis for single group before factor adjustment, CFA (θ3, 4 free; θ2, 3 free; (θ4, 5free) = single group factor analysis after adjusting intercepts of items 2 and 3; MGCFA (θ3, 4 free; θ2, 3 free; (θ4, 5free) = Multiple group confirmatory factor analysis with adjusted intercepts for items 2 and 3.

#### Measurement invariance across cultures (Multi-Group CFA)

**Figure 5:**
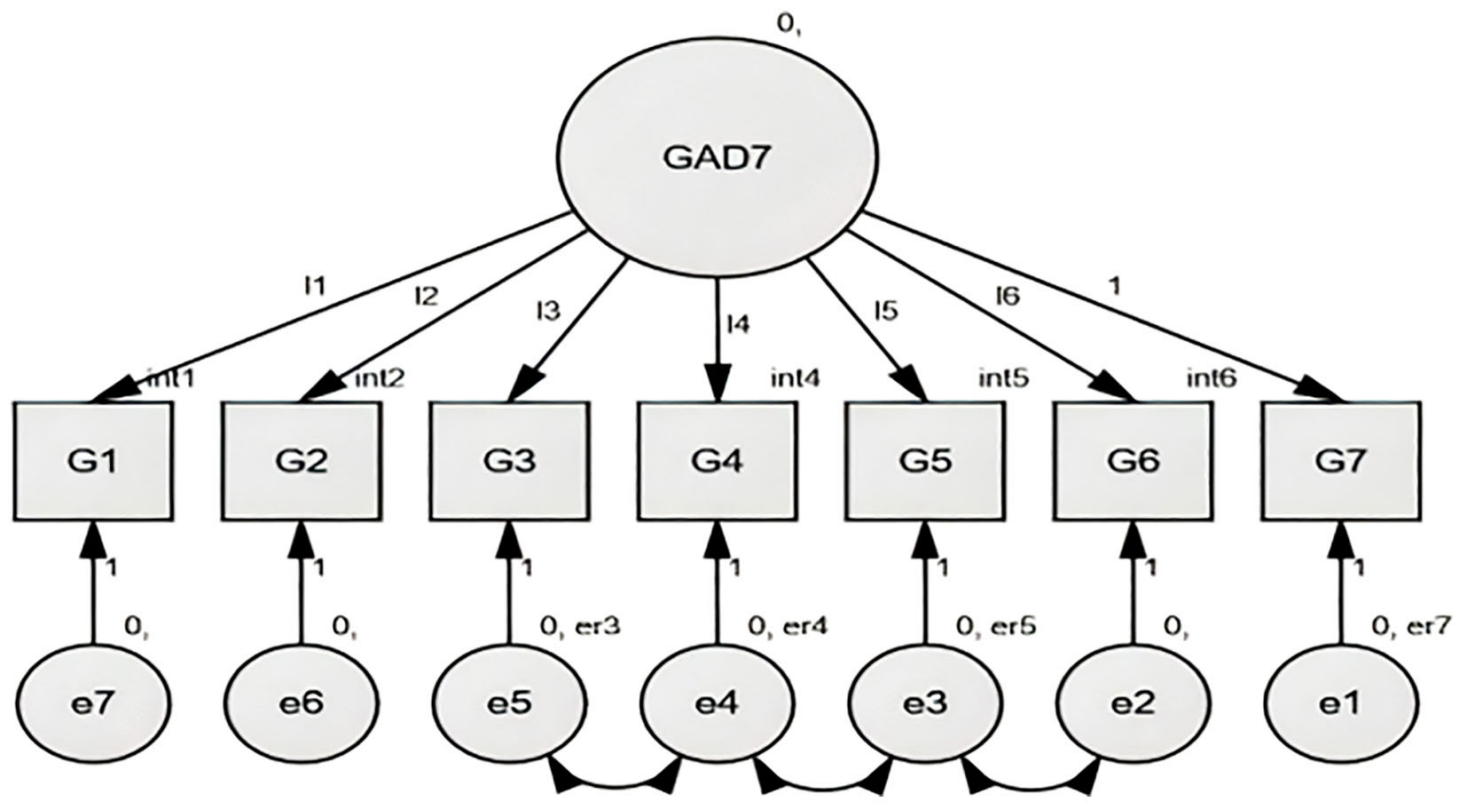
GAD-7 Factor structure Chinese and Rwandese adolescent students, the figure illustrates the factor loadings for each item of the GAD-7 in both cultural groups.

Configural invariance was established with excellent global fit (CFI = 0.989, RMSEA = 0.043, SRMR = 0.026, GFI = 0.987, TLI = 0.979, NFI = 0.988). Metric invariance was also supported, with minimal changes in fit indices (ΔCFI= 0.008, ΔRMSEA= 0.006). However, scalar invariance was not confirmed due to a significant decrease in model fit (ΔCFI= 0.032, ΔRMSEA= 0.025). Modification indices indicate high intercepts for items 7 (“feeling afraid, as if something awful might happen”) and 3 (“excessive worrying”). Freely estimating these intercepts improved model fit (ΔCFI= 0.008, ΔRMSEA= 0.007), confirming partial invariance (**Figure 5**). The change in Comparative Fit Index (ΔCFI) and ΔRMSEA parameters followed Cheung’s recommendations suggesting that a ΔCFI < 0.01 and ΔRMSEA < 0.015 (25) indicating a non-significant deterioration of the model fit sustaining invariance. The ΔCFI and ΔRMSEA scores for configural and metric invariance in this study were within these ranges, indicating that the GAD-7’s factor loadings and structure are comparable for Chinese and Rwandan adolescents, however at the scalar invariance level there were significant changes in fit indices.

#### Latent mean comparison

Latent mean comparisons revealed a non-significant trend towards higher anxiety levels in Rwandan adolescents compared to Chinese adolescents (z= 0.02, d= 0.033, P = 0.98).

## Discussion

This study evaluated the cross-cultural validity of the Generalized Anxiety Disorder-7 (GAD-7) scale among Chinese and Rwandan adolescents. While the GAD-7 demonstrated good psychometric properties and a consistent one-factor structure across both groups, only partial scalar invariance was achieved. This suggests that the GAD-7 is reliable for assessing anxiety symptoms within each cultural context but may not support direct comparisons of latent mean anxiety scores due to potential measurement biases influenced by cultural and linguistic factors.

The GAD-7 maintained a one-factor structure in both groups, indicating its ability to capture generalized anxiety similarly across these populations. However, cultural differences in the interpretation and expression of anxiety symptoms may affect the scale’s performance (27). For instance, the higher RMSEA values observed in the Chinese sample and the influence of cultural factors in Rwandan population highlight the variability in how anxiety is conceptualized and reported across cultures (28). The partial scalar invariance further underscores that while the GAD-7 can effectively detect anxiety symptoms, the severity of these symptoms may not be directly comparable between Chinese and Rwandan adolescents.

Notably, significant variations in item-level functioning were identified, particularly for items related to excessive worrying (“Worrying too much about different things”, item 3) and fear (“Feeling afraid, as if something awful might happen”, item 7), confirmatory factor analysis modification indices indicated that due to theoretical and cultural bases, these items exhibited differential item functioning (DIF) across both cultural groups implying variations in cultural interpretation, perception and expression of anxiety symptoms. For example, Rwandan adolescents may report higher levels of anxiety due to the integrational trauma and societal stressors associated with the country’s unique historical and sociocultural context, including the legacy of the 1994 genocide and ongoing socioeconomic challenges (29). In contrast, Chinese adolescents may express anxiety through somatic symptoms like headaches or physical tension, rather than emotional distress, reflecting cultural tendencies to avoid direct expressions of psychological vulnerability (19). Additionally, Chinese individuals with anxiety often exhibit shame-prone tendencies and concerns about social evaluations, which may further influence symptom reporting (30).

The contradiction between non-significant latent mean comparison analyses and significantly higher overt GAD-7 scores in Rwandan adolescents compared to the Chinese sample can be attributed to measurement bias stemming from partial scalar invariance which implicates DIF discussed above leading to inflated overt scores in one group without correspondence in the latent construct differences (19). Furthermore, it implies that this may not reflect true differences but rather cultural variations in anxiety expression, reporting and response patterns. For instance, linguistic differences in translating psychological scales can significantly alter meaning of items and impact response patterns (14, 31). In Rwanda, one of the primary languages, Kinyarwanda, may lack direct equivalent terms for words like “anxiety”, leading individuals to express emotional distress through physical discomfort (32, 33). Similarly, in China, the linguistic structure of Mandarin and cultural tendencies toward somatic expression may result in anxiety being reported through somatic symptoms such as fatigue, sleep disturbances, or chest pain (10, 34). These findings emphasize the importance of considering cultural and linguistic contexts when interpreting anxiety symptoms and designing culturally adapted assessment tools (23).

The lack of complete scalar invariance has important implications for cross-cultural mental health research and practice. Differences in item functioning and response patterns may lead to challenges in establishing universal cutoff scores and interpreting results across cultures. For instance, the same GAD-7 score might represent varying degrees of anxiety severity in different cultural settings due to differences in response patterns potentially leading to misdiagnosis or inappropriate treatment recommendations (13, 18). These findings highlight the need for culturally sensitive approaches to mental health assessment and the development of instruments that account for cultural variations in symptom expression to improve anxiety assessment accuracy and ensure that cutoff scores are culturally appropriate (16).

To improve mental health literacy and reduce stigma, education and healthcare systems must recognize somatic manifestations as potential indicators of underlying mental health issues. This study’s findings have several implications, first, healthcare professionals and educators should receive training in culturally sensitive approaches to mental health care, including the identification of culturally specific idioms of distress and the adaptation of assessment tools to local contexts. Translation efforts should prioritize contextual adaptability to ensure accurate symptom identification and conveyance; second, clinicians and researchers may need to adjust cutoff scores to provide culturally specific cut-off scores for the GAD-7, this can be addressed by adjusting setting a slightly cutoff score for populations where anxiety is expressed more for instance, also identifying culturally different items and adjusting the scoring weights by removing or modifying them. Finally, incorporate additional measures such as culturally adopted scales to enhance the accuracy of assessment in cross-cultural settings and provide a comprehensive understanding of anxiety in different populations. These measures may improve the cross-cultural validity of this assessment tool and ensure that patients receive appropriate health care.

While this study contributes valuable insights into the cross-cultural validity of the GAD-7, several limitations should be noted. First, the sample was drawn from a single city in each country, which may limit the generalizability of the findings and lead to regional biases due differing cultural and socioeconomic factors (3).While the samples were diverse within their respective cities they may fall short on capturing the broader picture of cultural and socioeconomic variations in both countries, thus future studies should include samples from multiple regions to enhance statistical power and external validity of the results and provide a more comprehensive understanding of anxiety symptoms across diverse populations. Second, the challenge of establishing full scalar invariance affects the interpretation and generalizability of the results. Finally, the reliance on self-report measures may introduce response biases, such as social desirability or cultural norms influencing symptom reporting. Incorporating objective measures, such as clinician-rated assessments or behavioral observations, could mitigate these biases in future research.

In conclusion, while the GAD-7 is a reliable tool for detecting anxiety symptoms within specific cultural contexts, its ability to support direct cross-cultural comparisons of symptom severity is limited by cultural and linguistic differences in symptom expression and interpretation. These findings underscore the need for culturally sensitive instruments and approaches in mental health assessment. Future research should explore the impact of cultural factors on symptom expression and measurement sensitivity, utilizing culturally adapted instruments and more diverse samples to advance cross-cultural mental health research and practice.

## Data Availability

The raw data supporting the conclusions of this article will be made available by the authors, without undue reservation.
